# Traditional Herbal Formula Taeeumjowi-Tang (TJ001) Inhibits p53-Mutant Prostate Cancer Cells Growth by Activating AMPK-Dependent Pathway

**DOI:** 10.1155/2019/2460353

**Published:** 2019-05-05

**Authors:** Sooyeon Kang, Hyo In Kim, Yu-Jeong Choi, Seul Ki Lee, Ji Hye Kim, Chunhoo Cheon, Seong-Gyu Ko

**Affiliations:** ^1^Department of Science in Korean Medicine, Graduate School, Kyung Hee University, Seoul 02447, Republic of Korea; ^2^Department of Korean Medicine, Graduate School of Kyung Hee University, 26 Kyungheedae-ro, Dongdaemun-gu, Seoul 02447, Republic of Korea; ^3^Department of Preventive Medicine, College of Korean Medicine, Kyung Hee University, 26, Kyungheedae-ro, Dongdaemun-gu, Seoul 02447, Republic of Korea

## Abstract

Dysregulated lipid metabolism is a prominent feature of prostate cancers (PCas); several enzymes involved in lipid accumulation are highly expressed. Here, we elucidated efficacy of TJ001, a traditional herbal decoction, in inhibiting* de novo* lipogenesis. TJ001 had significant cytotoxicity against DU145 but not PC3 and LNCaP cells and, similarly, TJ001 markedly AMPK phosphorylation only in DU145 cells. This was accompanied by the downregulation of phosphorylated-acetyl coenzyme A carboxylase (ACC) expression and sterol regulatory element-binding protein 1 (SREBP1) proteolytic cleavage, thereby inhibiting its role as a transcription factor to induce lipid biosynthesis. When Oil Red O staining was performed, it is reflected in the reduction of lipid droplets (LDs). TJ001 also induced G_1_/S cell cycle arrest* via *a cell cycle inhibitor (CKI) p21^WAF1/CIP1^ upregulation. Although p53 proteins remained unchanged, both cyclin E and cyclin D1 were decreased. Moreover, TJ001 suppressed the mammalian target of rapamycin (mTOR) signaling pathway. Generally, the prolonged G_1_/S phase arrest accompanies apoptosis, but TJ001 failed to work as a trigger apoptosis in DU145 cells. We showed that mutant p53 proteins were required for the survival of DU145 cells. In presence of TJ001, inhibition of endogenous mutant p53 by RNAi led to cell viability reduction and induction of the p-AMPK/AMPK ratio. In addition, it induced apoptotic cell death in DU145 cells. At the cellular level, induction of PARP, caspase-3, and caspase-9 cleavages was observed, and caspase-3 activity was increased in the p53 knockdown cells treated with TJ001. Taken together, we demonstrated that TJ001 inhibited cell growth in DU145 prostate cancer cells as indicated by blocking lipogenesis and induction in G_1_/S cell cycle arrest. In addition, we may provide an evidence that mutant p53 protein has potential role as an oncogenic action in DU145 cells. Collectively, the combination of mutant p53 targeting and TJ001 treatment resulted in decreased cell growth in DU145 cells.

## 1. Introduction

PCa is a major disease that has fatal effects on men's health around the world. This cancer represents not only the highest incidence of newcomers in males (180,890 estimated cases in 2018, 21.50%) but also second highest deaths (26,120 estimated cases in 2018, 8.31%) in the USA [1]. With the advent of the prostate-specific antigen (PSA) testing method, the overall PCa incidence declines steadily, but still accounts for a large part of the mortality in male [2]. Therefore, additional approaches and strategies are needed to the treatment of PCa. Studies to understand the specific lipid metabolism of PCa are still lacking.

One important mutation which often occurred in prostate cancer (PCa) is a tumour suppressor p53 [3]. The function of p53 is not limited to tumour suppression through cell cycle arrest and/or apoptosis, but it is also involved in cell metabolism, autophagy, and cell senescence [4–8]. If mutation occurs in p53, it does not work at all as well as wild-type p53 or exert dominant-negative effects over the remaining wild-type allele [9, 10]. Among them, some mutant p53 have the characteristic of tumour oncogenic action that promotes cell growth and progression [11–13]. However, the gain-of-function (GOF) of mutant p53 has not yet been fully understood. Therefore, it is valuable to understand role of mutant p53 in cancer cells and to discovery of the drug targeting mutant p53 in PCa as well as many kinds of cancer.

Dysregulated lipid metabolism is a prominent feature of PCa [14, 15]; cell anabolism, an adenosine triphosphate- (ATP-) consuming process that constructs macromolecules building up for cell growth, adopts* de novo* lipogenesis and catabolism, which apposite function to anabolism, and adopts *β*-oxidation using fatty acids (FAs). 5′ adenosine monophosphate-activated protein kinase (AMPK) is an enzyme that senses cellular energy status, largely to activate catabolic pathway and inhibit anabolic pathway when cellular energy is low [16]. This role for AMPK is not different in PCa [17, 18]. Once activated, AMPK triggers off the inhibition of* de novo* lipogenesis by targeting downstream metabolic enzymes acetyl-CoA carboxylase (ACC) and by phosphorylating sterol regulatory element-binding protein 1 (SREBP1) [19, 20].

ACC is a key enzyme in that converts acetyl-CoA to malonyl-CoA. The phosphorylation of ACC at Ser79 by AMPK activation prevents malonyl-CoA from being used as a substrate for fatty acid biosynthesis [21]. SREBP is a major transcription factor that regulates lipid metabolism and energy storage through the synthesis and absorption of fatty acids, triglycerides, and cholesterol [22]. It has also been reported that it is associated with aberrant lipid metabolism required for tumour growth [23]. AMPK suppresses SREBP1 proteolytic cleavage and represses SREBP1 target gene expression leading to lipogenesis and lipid accumulation [24].

Taeeumjowi-tang (TJ001) is a traditional Korean medicine that usually prescribed for a particular (Tae-eum) type of person to regulate stomach-related symptoms. TJ001 consists of eight herbal ingredients, listed in Table 1. In clinical practice, TJ001 is used especially for the obese patients, and the weight loss effects of TJ001 have been revealed through some clinical studies [25]. However, until recently, it has never been applied as a treatment for cancer. In the present study, we investigated that anticancer effects of TJ001 on PCa cells and its mechanisms of action on lipid metabolism-related proteins expression.

## 2. Materials and Methods

### 2.1. Chemicals and Reagents

Taeeumjowi-tang (TJ001) extracts were provided by Hanpoong Pharm & Foods Co., Ltd. (Republic of Korea, K-GMP). All media for cell culture were from Welgene (Inc., Republic of Korea). Fetal Bovine Serum (FBS) was obtained from J R Scientific (Inc., USA). Annexin-V-FITC was purchased from BD Biosciences (USA). Compound C, 7-Aminoactinomycin D (7-AAD) and *α*-tubulin antibody was from Sigma-aldrich (USA). SREBP1 and *β*-actin antibody was purchased from Santa cruz (Inc., USA). Antibodies against p-AMPK*α*, AMPK*α*, p-ACC, ACC, p53, p21, p27, cyclin E, cyclin D1, p-S6K, S6K, p-4EBP1, 4EBP1, PARP, cleaved caspase-3, caspase-8, caspase-9, and GAPDH were from Cell Signaling Technology (Inc., USA).

### 2.2. Cell Lines and Cell Culture Conditions

All human prostate cancer cell lines were obtained from the American Type Culture Collection (ATCC, Inc., USA). DU145 (p53 mutant), PC3 (p53 null) and WI38-V/A13 cell lines were cultured in RPMI 1640 supplemented with 10% heat-inactivated FBS and 1% penicillin-streptomycin. LNCaP (p53 wild-type) cells were grown in RPMI 1640 with 10% heat-inactivated FBS, 1% penicillin-streptomycin, 4500 mg/L D-glucose, 2 mM L-glutamine, 10 mM HEPES, 1 mM sodium pyruvate, and 1500 mg/L sodium bicarbonate. RIE cells were grown in DMEM containing 10% heat-inactivated FBS and 1% penicillin-streptomycin. MCF10A cells were maintained in DMEM containing 5% horse serum, EGF 100 *μ*g/mL, hydrocortisone 10 mg/mL, cholera toxin 1 mg/mL, insulin 10 mg/mL, and 1% penicillin-streptomycin. Cell lines were incubated at 37°C in a humidified atmosphere with 5% CO_2_.

### 2.3. Cell Viability Assay

The cell viability was assessed using MTT assay. Cells were seeded in 5 × 10^3^ cells per well in 96-well plate and grown for 24 h. Cells were exposed to indicated concentrations of TJ001 (25, 50, 100, and 200 *μ*g/mL) for analysis. TJ001 was treated for 48 h, aqueous MTT solution was then added to 20 *μ*L each well, and the mixture was incubated at 37°C for 2 h. At the end of 2 h add 100 *μ*L of The lysis buffer 20% w/v of SDS is dissolved at 37°C in a solution comprising 50% DMF (N,N-dimethyl formamide) in D.W. per well and incubated at 37°C for overnight. These samples were measured with a 96-well ELISA plate reader (Merck, Germany) at 590 nm test wavelength.

### 2.4. Formation of Colony Assay

The ability of colony formation was measured by the clonogenic assay [26]. Cells were plated at 6-well plate at a density of 5 × 10^2^ cells per well. After 24 h stabilization, cells were grown on RPMI 1640 supplemented with TJ001 (200 *μ*g/mL) for additional 7 days until colony formation was generated. The colonies were stained with 0.25% crystal violet.

### 2.5. WST-1 Assay

After indicated treatment time, the WST-1 reagent (Daeillab, Republic of Korea) was added in multiwell plates and incubated for up to 2 h before reading the plate. This sample was quantified by measuring the absorbance at 450 nm using an ELISA reader.

### 2.6. ATP Detection Assay

ATP level was evaluated by Mitochondrial ToxGlo™ Assay (Promega, USA) according to the manufacture's protocol. Briefly, DU145 cells were seeded in white clear bottom 96-well culture plate (Thermo Fisher, USA). Treated cells were incubated at 37°C in a humidified and CO_2_-supplemented incubator for 48 h. ATP detection reagent (100 *μ*L) was added to the multiwell plate and then mixed for 3 min. Luminescence was measured using a Fluoroskan FL Microplate Fluorometer, and Luminometer (Thermo Fisher, USA).

### 2.7. Oil Red O Staining Assay

After removal of the culture medium, cells were washed gently with twice DPBS and fixed in 10% formalin for 10 min at room temperature. Then, cells were replaced with fresh 10% formalin for 2 h. After isopropanol wash, for Oil Red O staining, fixed cells were treated with ORO stain (0.5% ORO in 100% isopropyl alcohol, diluted with distilled water in the ratio of 3:2) for 30 min. After removal of free dyes by distilled water washing, cells were captured under a microscope (Olympus, Japan).

### 2.8. Transient Transfection Assay

Transfections were carried out by using Lipofectamine RNAi max (Invitrogen, USA) according to the manufacturer's instructions. In brief, DU145 cells with 10 nM of control or p53 siRNA were incubated in Opti-MEM® Reduced Serum Medium (Thermo Fisher, USA) for 24 h and treated with TJ001 in fresh medium 48 h. The protein expression of p53 in targeted cells was monitored by SDS-PAGE and immunoblot assay.

### 2.9. Cell Cycle Analysis by Propidium Iodide (PI) Staining

DU145 cells were exposed to the indicated agent for 48 h. Cells (1 × 10^5^) were washed twice with DPBS, added to 0.8 mL DPBS, and gently resuspended by 2.2 mL 95% ethanol. Fixing buffer was removed for collecting the cell, and the remaining cells were centrifuged at 1,500 rpm for 5 min at 4°C. Propidium iodide (1 mg/mL) in DPBS was added to the cells for 30 min at room temperature. After 30 min, cells were filtered through 40 *μ*m cell strainer. Stained cells were acquired by a FACSalibur flow cytometer (BD Biosciences, USA), and then analysis was executed using CellQuest™ Pro (BD Biosciences, USA).

### 2.10. Cell Apoptosis Analysis by Annexin-V-FITC and 7-AAD Staining

Human prostate carcinoma DU145 cells were cultured in RPMI 1640 medium at a density of 1 × 10^5^ cells per 60 mm dish. Cells were maintained with TJ001 (200 *μ*g/mL) treatment for 48 h and collected at indicated time points. Collected cells were washed with DPBS, stained with annexin-V-FITC and stained with 7-AAD for detecting apoptotic cells. Stained cells were analyzed by using a FACS flow cytometer.

### 2.11. Immunoblot Analysis

Harvested cells were lysed with RIPA buffer (Biosesang, Republic of Korea) and incubated in 4°C for 30 min. Cell lysates were centrifuged at 13,000 rpm, 4°C for 20 min to remove insoluble materials. The protein concentrations were determined by using the Bio-Rad protein assay dye (Bio-Rad Laboratories, USA). Protein extracts were mixed with 5X Laemmli Sample Buffer (LSB) (62.5 mM Tris-HCl [pH 6.8], 25% glycerol, 2% SDS, 2-mercaptoethanol, 0.01% bromophenol blue, D.W.) and denatured by heating to 100°C. An equal amount of protein was separated on % SDS-PAGE gels. The proteins were then transferred to a nitrocellulose membrane (Amersham, USA). The bands were developed by the ECL detection kit (DoGEN, Seoul, Korea).

### 2.12. Caspase-3 Activity Assay

Caspase-3 activity was determined using the caspase-3/CPP32 Colorimetric Assay kit (BioVision, Inc., USA), according to manufacturer's protocol. Briefly, samples were mixed with 2X Reaction Buffer containing 10 mM DTT and 4 mM DEVD-*p*NA substrate, followed by dilution with Cell Lysis Buffer, setting a volume of 100 *μ*L/sample in a 96-well plate. Caspase activity was measured at 405 nm using a 96-well ELISA plate reader.

### 2.13. Statistical Analysis

Quantitative data are expressed as means ± standard deviation (SD). Significance was assessed by t-test using Microsoft Excel 2010 software.* P* value was considered as significant differences (∗*p*<0.05, ∗∗*p*<0.01, and ∗∗∗*p*<0.001).

## 3. Results

The primary objective of this study was to determine whether TJ001 can exert anticancer effects through lipid metabolism-related pathway on PCa cells.

### 3.1. TJ001 Treatment Decreases Viability and Proliferation in Prostate Cancer Cells

**Figure 1:**
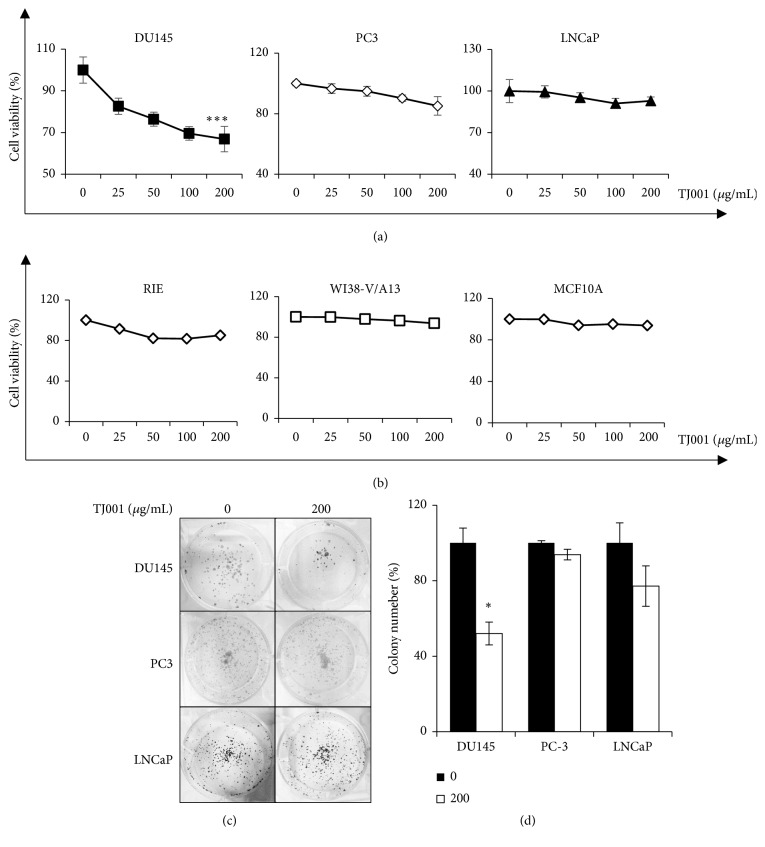
TJ001 inhibits cell growth in prostate cancer cells. (a) The regulation of prostate cancer (p53 mutant DU145, p53 null PC3, and p53 wild-type LNCaP) cells proliferation by TJ001. Cell viability was measured by the MTT assay; optical density of MTT solution was measured at 590 nm. Data represent the average of three independent experiments [error bars are standard deviation (SD) (∗∗∗*p* < 0.001)]. (b) Cell viability after TJ001 treatment in normal cells. (c) Clonogenic ability of DU145, PC-3 and LNCaP cells after TJ001 treatment. Cells were treated with or without 200 *μ*g/mL TJ001 for 7 days. The formed colonies were stained using 0.25% crystal violet. [Left panel; clonogenic plates with 0 *μ*g/mL, right panel; clonogenic plates with 200 *μ*g/mL TJ001.] (d) The graph showed the number of colonies in (c). Error bar represents SD (∗*p* < 0.05).

To investigate the effects of TJ001 on the cell proliferation of PCa cells, all cancer cell lines were treated with varying concentrations of TJ001 (0, 25, 50, 100, and 200 *μ*g/mL) for 48 h. Cell viability was determined using MTT assay. As seen in Figure 1(a), DU145 cells showed the highest decrease rate to cell viability (66.85%) at TJ001 (200 *μ*g/ml) treatment. DU145 cells were susceptible to TJ001 compared with those PC3 (85.15%) or LNCaP (92.93%) cells. In addition, TJ001 had no effects in normal cells (Figure 1(b)). Clonogenic ability is important to a single cell to grow into a colony for unlimited division in cancer. Therefore, the influence of TJ001 of colony formation in PCa cells was seen in Figure 1(c). TJ001 induced a decline in formation of colonies, with the 200 *μ*g/ml concentration inhibiting the DU145 cell growth to 52.05 ± 2.58%∗ (∗*p*<0.05) of that in the control cells during 7 days (Figure 1(d)). These results suggest that TJ001 induced inhibition of cell growth in particular PCa type, DU145.

### 3.2. TJ001 Impedes Lipid Accumulation through AMPK Pathway Activation

**Figure 2:**
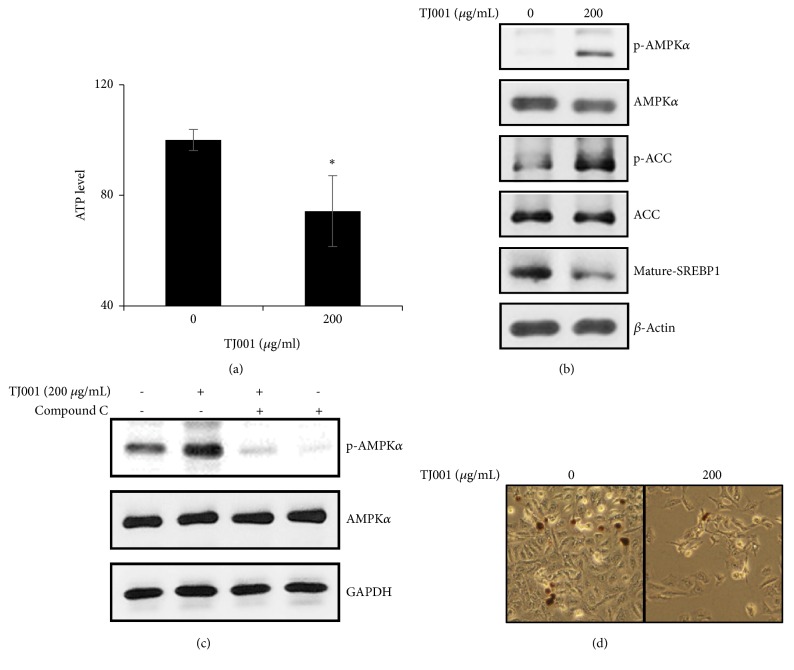
Inhibitory effects of TJ001 on lipid accumulation in DU145 cells. Cells were incubated for 48 h with or without TJ001 (200 *μ*g/mL). (a) The content of ATP was measured using a commercial kit (Promega, USA). Data are presented as the mean ± SD (∗*P* < 0.05 compared with the control). We analyzed (b) the expression of lipid metabolism-related proteins, (c) the effects of compound C (c.c) on phosphorylated AMPK (p-AMPK). (d) Lipid accumulation was visualized using an Olympus CKX41 inverted microscope at ×300 magnification [left panel; Oil Red O stained cells with 0 *μ*g/mL, right panel; with 200 *μ*g/mL TJ001].

Since TJ001 was originally used as a treatment for obesity, it would affect the metabolism of PCa using fatty acids (FAs) and cholesterols [27]. Therefore, we investigated whether TJ001 regulates mitochondrial ATP product. In the presence of TJ001, we determined mitochondrial ATP product was decreased in DU145 cells (∗p<0.05) (Figure 2(a)), but not PC3 and LNCaP cells (Supplementary 1(a)). AMPK, a highly conserved master regulator of energy homeostasis, responds to metabolic stress at both the cellular and physiological levels. We observed the induction of AMPK phosphorylation due to energy imbalance. In addition, there was activity of ACC and SREBP also decreased (Figure 2(b)), but not PC3 and LNCaP cells (Supplementary 1(b)). To confirm AMPK activation performed by TJ001 treatment, DU145 cells were incubated with pretreated compound C, a competitive inhibitor of AMPK (Figure 2(c)). Next, we assessed the effects of TJ001 on lipid accumulation by Oil Red O (ORO) staining that stains neutral lipid content (Figure 2(d)). Treatment with 200 *μ*g/mL TJ001 led to a decrease in lipid accumulation compared to control. These results indicate that TJ001 showed a change in ATP level, which led to AMPK activation and also to inhibition of lipid accumulation.

### 3.3. TJ001 Induces *G*_1_/S Cell Cycle Arrest

**Figure 3:**
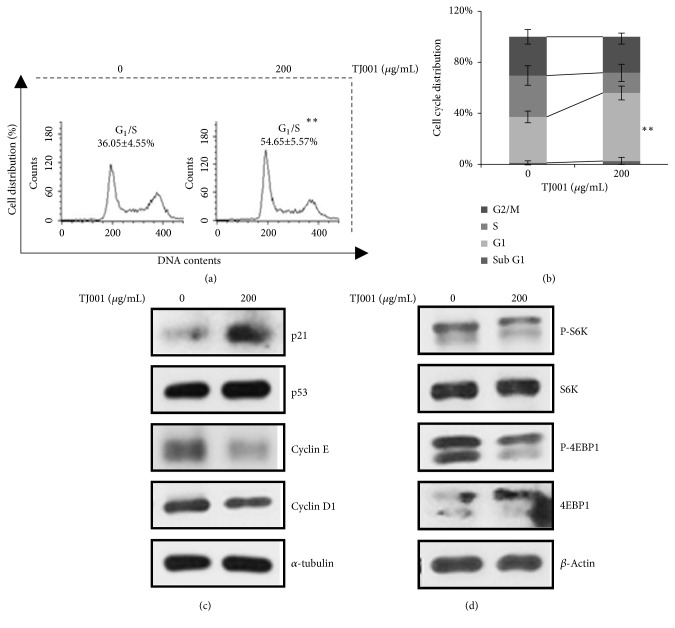
TJ001 induces G1/S phase arrest in DU145 cells. Cell cycle distribution of DU145 cells measured by flow cytometry. (a) Cells were incubated with TJ001 (200 *μ*g/mL) treatment for 48 h. All floating and attached cells were harvested and fixed in 95% ethanol. The stained cells with PI were performed to examine cell cycle progression (b) The graphs showed a cell cycle distribution in DU145 prostate cancer cells. Data represents mean ± SD (∗∗* p*< 0.01) (c) CKI (Cyclin kinase inhibitor) and cyclin proteins, involved with G1/S phase arrest, were performed. (d) Total and phosphorylated forms of protein synthesis-related proteins in mTOR signaling pathway were detected using immunoblotting assay.

The cell proliferation, regardless of the period, was affected by whether cell cycle progression works properly or not. The cell cycle distribution was examined using FACS analysis with PI staining. In DU145 cells, the cell cycle profiles resulted in no change in Sub G_1_ phase (from 1.12 ± 1.61% to 2.66 ± 2.89%), a decrease of cells in S phase (from 32.40 ± 7.64% to 16.2 ± 6.88%), and a slight decrease of cells in G_2_ phase (from 30.43 ± 5.79% to 28.96 ± 5.86%) and G_1_ phase was statistically increased up to 54.65 ± 5.57%∗ (∗∗*p*<0.01) (Figures 3(a) and 3(b)). However, PC3 and LNCaP did not show significant results (Supplementary 2). The results revealed that TJ001 has antiproliferation activity in DU145 cells through G_1_/S cell cycle arrest.

### 3.4. TJ001 Controls Cell Proliferation* via* Cell Cycle Regulatory Proteins and in AMPK-Dependent Manner

In order to validate the mechanism in cellular level by which TJ001 induced G_1_/S cell cycle arrest, we examined the expression level of key regulator involved in the G_1_/S checkpoint. Cdk4/6-Cyclin D1 and Cdk2-Cyclin E complex is required for the progression to S phase of the cell cycle that determines initiation of DNA replication [28]. Although p53 expression remained unchanged, treatment of DU145 cells with 200 *μ*g/mL TJ001 not only increased the level of p21^WAF1/CIP1^ proteins but also decreased the expression level of cyclin E and cyclin D1 (Figure 3(c)) [29]. TJ001 induced AMPK activation, which is considered to be linked to the mammalian target of rapamycin (mTOR) signaling implicated in cell growth [30]. Further investigation of mTOR signaling pathways showed that TJ001 suppressed phosphorylation of p70S6 kinase (p70S6K) at Thr389, and eukaryotic initiation factor 4E binding protein 1 (4EBP1) at Ser65. (Figure 3(d)). These proteins work as a hallmark of activation by mTOR and correlated with protein synthesis [31]. These experiments showed no inhibitory effects on PC3 and LNCaP cells (Supplementary 2). Therefore, these results demonstrate that TJ001 inhibits cell cycle progression and protein synthesis only in DU145 alone.

### 3.5. Effects of TJ001 on Apoptotic Cell Death

**Figure 4:**
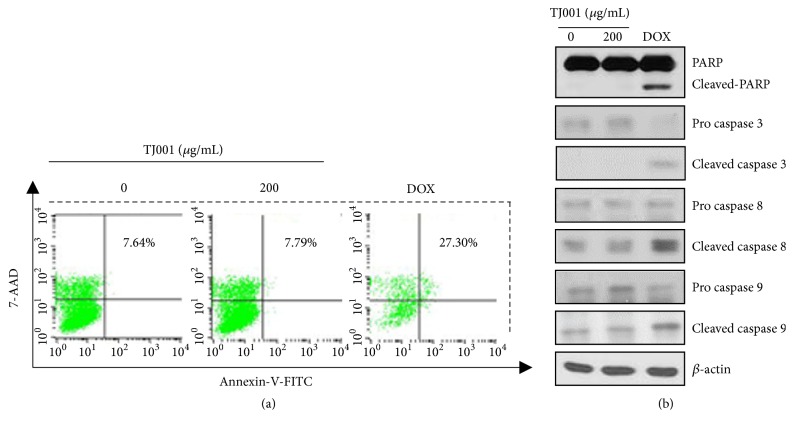
TJ001 had no effects on apoptosis in DU145 cells. (a) Cells (1X10^2^) were grown with or without TJ001 (200 *μ*g/mL) in 60pi dish for 48 h. Doxorubicin (DOX) was also used as a positive control. Harvested cells were stained with annexin-V-FITC for detecting apoptosis, then were stained with 7-AAD for detecting DNA contents for 15 min each. Cell apoptosis was determined by FACS analysis. (b) Apoptosis-regulated protein levels were evaluated after TJ001 (200 *μ*g/mL) treatment. Anti-*β*-actin was a loading control.

The increase in the G_1_/S phase may enhance a damaged cell to undergo apoptosis [32]. To elucidate the ability of TJ001 on the trigger apoptosis of PCa cells, samples were analyzed by FACS with annexin-V-FITC and 7-AAD staining. Doxorubicin, a widely used chemotherapy agent, acted as the positive control. The data analysis revealed that TJ001 had no increase the apoptotic rate, in comparison with doxorubicin that increased apoptosis in DU145 cells (Figure 4(a)). To determine whether cell apoptosis arise in cellular level, we examined the effects of TJ001 on the expression of PARP, caspase-3, caspase-8, and caspase-9 proteins. As shown in Figure 4(b), Apoptosis-involved factors appeared no change in TJ001 (200 *μ*g/mL)-treated cells. But, in doxorubicin-treated cells, the level of these proteins changed. The experimental results were negative for both PC3 and LNCaP cells (data not shown). This data demonstrated that TJ001 leads to no apoptotic cell death.

### 3.6. Knockdown of Mutant p53 Further Restrains Cell Growth and Induces Apoptosis

**Figure 5:**
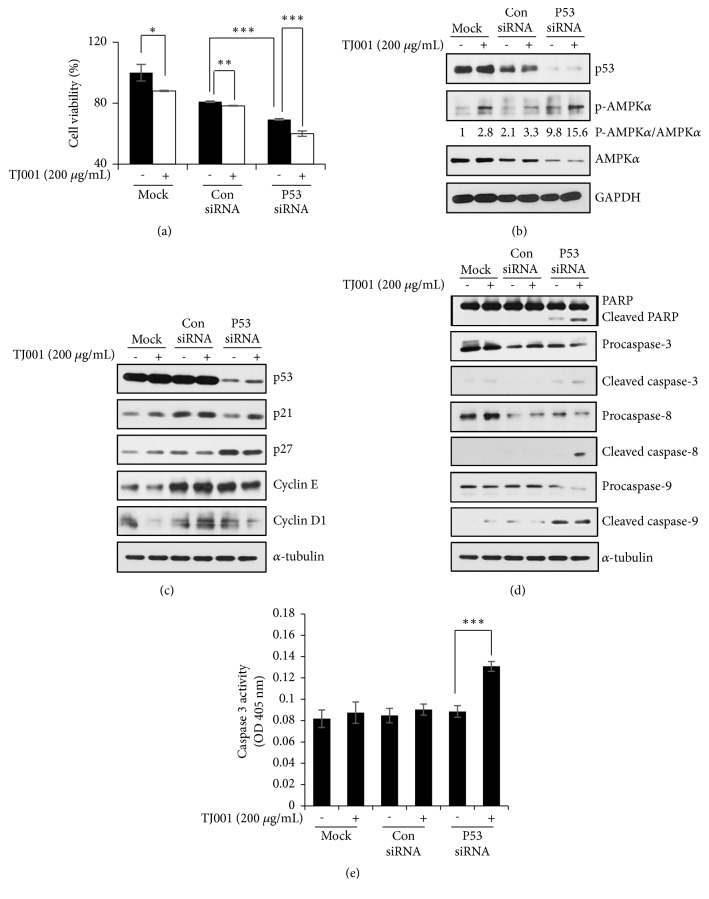
Effects of mutant p53 knockdown with TJ001 treatment in DU145 cells. After cells were transfected with p53 siRNA or control siRNA, and then cells were treated with TJ001. (a) The cell viability was evaluated by WST-1 assay. (∗*P*<0.05, ∗∗*P*<0.01, and ∗∗∗*P*<0.001). The total lysates were assessed by western blot analyses. Following to transfection, (b) expression of p-AMPK/AMPK ratio, (c) levels of cell cycle-related proteins expression and (d) PARP, capsase-3, capsase-8, and capsase-9 were measured. (e) Quantitative analysis of caspase-3 activity* in vitro*. Cell proteins were diluted in Cell Lysis Buffer and assayed for caspase-3 activity using DEVD-*p*NA as substrate. Results were measured at 405 nm wavelength (∗∗∗*p*<0.001).

The genomic* TP53* status of DU145 (p53 mutant), PC3 (p53 null), and LNCaP (wild-type p53) PCa cell lines had been reported [33]. From the previous data, the influence of TJ001 was valid only in DU145 cells. Then, we focused on gain-of-function of p53 mutation in DU145 cells. We examined the effects of mutant p53 knockdown on cell survival in DU145 cells. As shown in Figure 5(a), cell viability was significantly reduced by silencing p53 with RNAi, and TJ001 treatment was further reduced than nontreated p53 knockdown cells. Recently, mutant p53 was shown to conflicting with the activation of AMPK [34]. Thus, we examined whether AMPK activation was affected by knockdown of mutant p53. When mutant p53 was silenced, p-AMPK/AMPK ratio was increased with or without TJ001 (Figure 5(b)). Then we performed to elucidate how the preceding results were applied to the regulation of cell growth. The impact of silencing mutant p53, in treatment with TJ001, on cell cycle was examined by immunoblot analysis (Figure 5(c)). Compared with the mock and control siRNA transfections, in the presence of TJ001, p53 knockdown cells resulted in an upregulation in p21^WAF1/CIP1^ protein expression. Since depletion of mutant p53 gene by RNAi inhibited* in vitro* growth of DU145 cells, we further assessed whether gene-silencing of p53-induced growth inhibition was able to affect apoptotic cell death. As shown in Figure 5(d), gene-silencing of p53 under TJ001 treatment induced cleavage of PARP, caspase-3, caspase-8, and caspase-9 in DU145 cells. To further confirm whether mutant p53 knockdown could lead to apoptotic cell death in the presence of TJ001, we assessed caspase-3 activity in the lysates of the cells. Exposure to TJ001 to p53 silencing cells resulted in markedly increased activity of caspase-3 (Figure 5(e)). This result suggests that the mutation of p53 suppresses AMPK activation and contributes to DU145 cell proliferation. Collectively, when mutant p53 genetic interference occurs, it becomes more sensitive to TJ001 treatment.

## 4. Discussion

In traditional Korean medicine, TJ001 is a prescription for treating exterior cold symptoms of Tae-eum type of person. TJ001 has recently been reported to be effective in several studies related to obesity [35]. For example, case reports have already demonstrated weight loss, inhibition of hepatic lipid accumulation, and animal studies have reported antihyperlipidemic effects [36, 37]. Based on these studies, it was proposed that TJ001 would exhibit a beneficial effect in attenuating disturbance of lipid metabolism. However, the effect of TJ001 has not yet been sufficiently proven to treat cancer. Thus, in the present study aimed to the effects of TJ001 on prostate cancer cells (DU145, PC3, and LNCaP) with aberrant lipid metabolism. We found that the traditional Korean herbal formula TJ001 inhibited cell viability and proliferation in DU145 but not PC3 and LNCaP cells.

AMPK is a serine/threonine protein kinase that modulates cellular metabolism and energy balance. When the cellular energy required for cell survival is decreased, AMPK is activated and downstream signal pathway also regulated to inhibit* de novo* lipogenesis, which belongs to cell anabolism. In the current study, we observed that cellular ATP levels were decreased and phosphorylation of AMPK was increased by treatment of TJ001 in DU145 cells. Consistent with the activation of AMPK, the phosphorylation of ACC was also increased and the mature SREBP1 was decreased after TJ001 administration, indicating that the AMPK pathway in DU145 cells was activated in the presence of TJ001. We used AMPK inhibitor compound C to confirm the actions of TJ001 on AMPK activation. As expected, the compound C pretreatment resulted in a significant decrease in TJ001-induced p-AMPK expression, suggesting that TJ001 could be an AMPK activator. The results showed that TJ001 treatment resulted in an attenuation in lipid accumulation which was visualized through Oil Red O staining.

As AMPK inhibits* de novo* lipogenesis, its mechanism diminishes lipid synthesis and blocks cell dividing and tumour growth [38]. The* in vitro* study further demonstrates that TJ001 suppresses G_1_/S cell cycle progression, accompanied by the downregulation of cyclin D1 and cyclin E and the upregulation of p21^WAF1/CIP1^. However, the expression of the p53 proteins was observed to be unchanged. The mTOR is AMPK downstream targets functions as a sensor to control protein synthesis for cell growth. Hence, TJ001 inhibited mTOR kinase activity by dephosphorylating downstream effectors (S6K, 4EBP1). Apoptotic cell death is one of the major cause for the inhibition to cell growth. While doxorubicin, a positive control, caused apoptosis, TJ001 had no effects in DU145 cells.

GOF mutant forms of p53 can induce an increase in carcinogenic properties that promote tumour growth and progression. As reported previously, the silencing of mutant p53 gene in DU145 cells* via* RNAi resulted in considerable inhibition of cell viability and growth, which associated with cell cycle arrest and apoptosis [39]. Therefore, it is evident that DU145 is highly dependent on mutant p53 for cell survival and proliferation. In the present study, the depletion of mutant p53 increased inhibition of cell growth in comparison to the mock and control siRNA group. The cell viability of p53 knockdown groups presented significantly degrees of decrease under TJ001 treatment. Tumours with mutant p53 rapidly grow by ingesting a lot of nutrients to fuel the survival signaling pathway, which cancer cells with p53 mutations may become more susceptible to metabolic stress. Since mutant p53 binds to AMPK and inhibits activation, we hypothesized that knockdown of mutant p53 induces AMPK activation [34]. Then, it was confirmed by western blotting assay to observe whether mutant p53 depletion affects AMPK activation. Knockdown of mutant p53 resulted in decreased inhibition of AMPK activation and increased the expression of p-AMPK/AMPK ratio when treated with TJ001, suggesting that the role of TJ001 as an AMPK activator was partially suppressed by mutant p53, and its activation was enhanced when mutant p53 was knockdown. Since our gene knockdown showed a decrease in cell growth, we analyzed its expression after cells were treated with TJ001. We observed p27^Kip1^ upregulation when mutant p53 knockdown without TJ001 treatment. In the presence of TJ001, we observed upregulation of p21^WAF1/CIP1^ and downregulation of cyclin E and cyclin D1 expression. As mentioned earlier, TJ001 treatment did not induce apoptosis in DU145 cells with functional mutant p53. However, in the cells transfected with p53 siRNA, the induction of apoptosis was observed in protein expression by PARP, caspase-3, caspase-8, and caspase-9 cleavages and caspase-3 activity was also significantly increased with TJ001 treatment. These results indicated that the combination of mutant p53 knockdown and TJ001 showed a greater impact on DU145 cell growth.

## 5. Conclusions

In conclusion, our results have demonstrated that TJ001 inhibited cell growth in DU145 (p53 mutant) prostate cancer cells as indicated by blocking lipogenesis and induction in G_1_/S cell cycle arrest. Furthermore, combining with mutant p53 targeting induced apoptotic cell death. Thus, TJ001 might act as a therapeutic agent for preventing hormone-resistant prostate cancer bearing p53 mutations.

## Figures and Tables

**Table 1:** Constituents of Taeeumjowi-Tang (TJ001) [36].

Herbal Formula	Name of herb	Amount (g)

Taeeumjowi-tang (TJ001)	*Semen Coicis*	3.75
	*Semen Castaneae*	3.75
	*Semen Raphani*	2.5
	*Schisandrae Fructus*	1.25
	*Liriopis tuber*	1.25
	*Herba Ephedrae*	1.25
	*Radix platycodi*	1.25
	*Acori Tatarinowii*	1.25
	*Rhizoma*	1.25

Total amount		17.5

## Data Availability

All data used to support the findings of this study are available from the corresponding author upon request.

## Supplementary Materials

Supplementary Materials Supplementary 1: Effects of TJ001 on metabolic stress in PC3 and LNCaP cells. Cells were incubated for 48 h with or without TJ001 (200 *μ*g/mL). (a) The content of ATP was measured using a commercial kit (Promega, USA). Data are presented as the mean ± SD. We analyzed (b) the expression of lipid metabolism-related proteins. Supplementary 2: TJ001 had no effects on cell growth in PC3 and LNCaP cells. Cell cycle distribution of prostate cancer cells measured by flow cytometry. (a) Cells were incubated with TJ001 (200 *μ*g/ml) treatment for 48 h. All floating and attached cells were harvested and fixed in 95% ethanol. The stained cells with PI were performed to examine cell cycle progression. (b) The graphs showed a cell cycle distribution in PC3 and LNCaP cells. Data represents mean ± SD. (c) CKI (cyclin kinase inhibitor) and cyclin proteins, involved with G1/S phase arrest, were performed. (d) Total and phosphorylated forms of protein synthesis-related proteins in mTOR signaling pathway were detected using immunoblotting assay.
