# Supplementary material for: Traditional Herbal Formula Taeeumjowi-Tang (TJ001) Inhibits p53-Mutant Prostate Cancer Cells Growth by Activating AMPK-Dependent Pathway
Source: Evid Based Complement Alternat Med. 2019 May 5;2019:2460353. doi: 10.1155/2019/2460353 (PMC6525874; doi:10.1155/2019/2460353)
Supplement: Supplementary Materials — Supplementary 1: Effects of TJ001 on metabolic stress in PC3 and LNCaP cells. Cells were incubated for 48 h with or without TJ001 (200 μg/mL). (a) The content of ATP was measured using a commercial kit (Promega, USA). Data are presented as the mean ± SD. We analyzed (b) the expression of lipid metabolism-related proteins. Supplementary 2: TJ001 had no effects on cell growth in PC3 and LNCaP cells. Cell cycle distribution of prostate cancer cells measured by flow cytometry. (a) Cells were incubated with TJ001 (200 μg/ml) treatment for 48 h. All floating and attached cells were harvested and fixed in 95% ethanol. The stained cells with PI were performed to examine cell cycle progression. (b) The graphs showed a cell cycle distribution in PC3 and LNCaP cells. Data represents mean ± SD. (c) CKI (cyclin kinase inhibitor) and cyclin proteins, involved with G1/S phase arrest, were performed. (d) Total and phosphorylated forms of protein synthesis-related proteins in mTOR signaling pathway were detected using immunoblotting assay. [file 2460353.f1.pdf]

Supplementary legend

Supplementary 1: Effects of TJ001 on metabolic stress in PC3 and LNCaP cells. Cells were incubated for 48 h with or without TJ001 (200 μg/mL). (a) The content of ATP was measured using a commercial kit (Promega, USA). Data are presented as the mean ± SD. We analyzed (b) the expression of lipid metabolism related proteins.

Supplementary 2: TJ001 had no effects on cell growth in PC3 and LNCaP cells. Cell cycle distribution of prostate cancer cells measured by flow cytometry. (a) Cells were incubated with TJ001 (200 μg/ml) treatment for 48 h. All floating and attached cells were harvested and fixed in 95% ethanol. The stained cells with PI were performed to examine cell cycle progression (b) The graphs showed a cell cycle distribution in PC3 and LNCaP cells. Data represents Mean ± SD (c) CKI (Cyclin kinase inhibitor) and cyclin proteins, involved with G1/S phase arrest, were performed. (d) Total and phosphorylated forms of protein synthesis-related proteins in mTOR signaling pathway were detected using Immunoblotting assay.

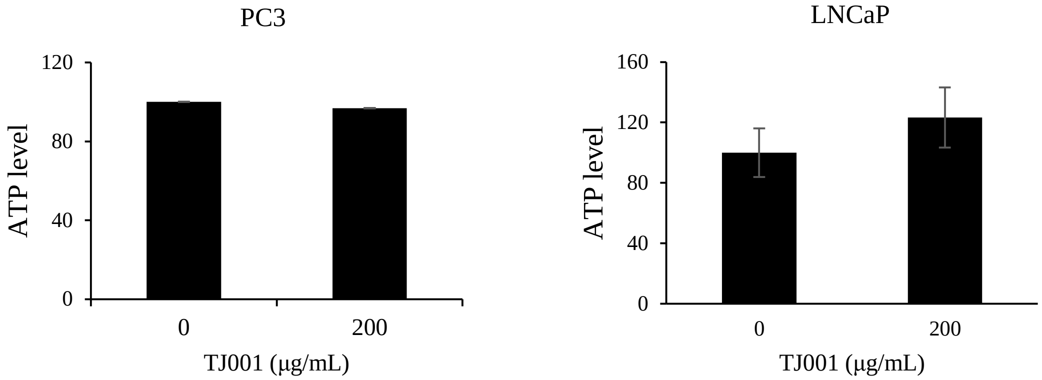
PC3
120
80
40
0
ATP level
0
200
TJ001 (μg/mL)
LNCaP
160
120
80
40
0
ATP level
0
200
TJ001 (μg/mL)

(a)

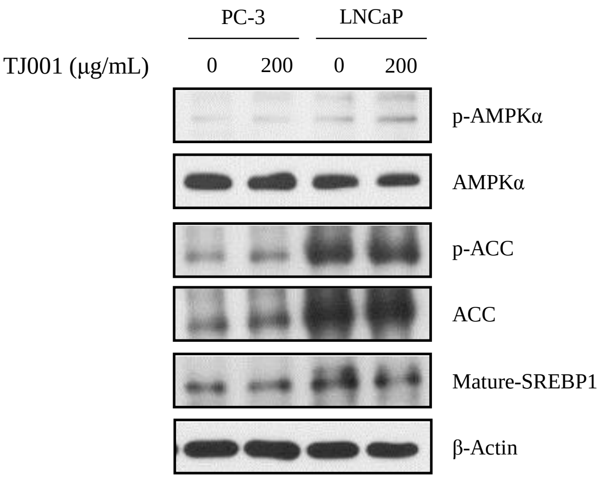
PC-3
LNCaP
TJ001 (μg/mL)
0
200
0
200
p-AMPKα
AMPKα
p-ACC
ACC
Mature-SREBP1
β-Actin

(b)

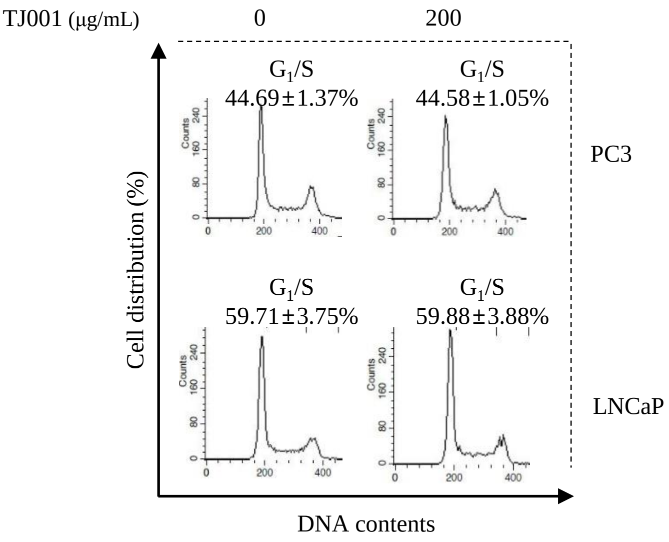
TJ001 (μg/mL)
0
200
G1/S
44.69±1.37%
G1/S
44.58±1.05%
PC3
G1/S
59.71±3.75%
G1/S
59.88±3.88%
LNCaP
Cell distribution (%)
DNA contents

(a)

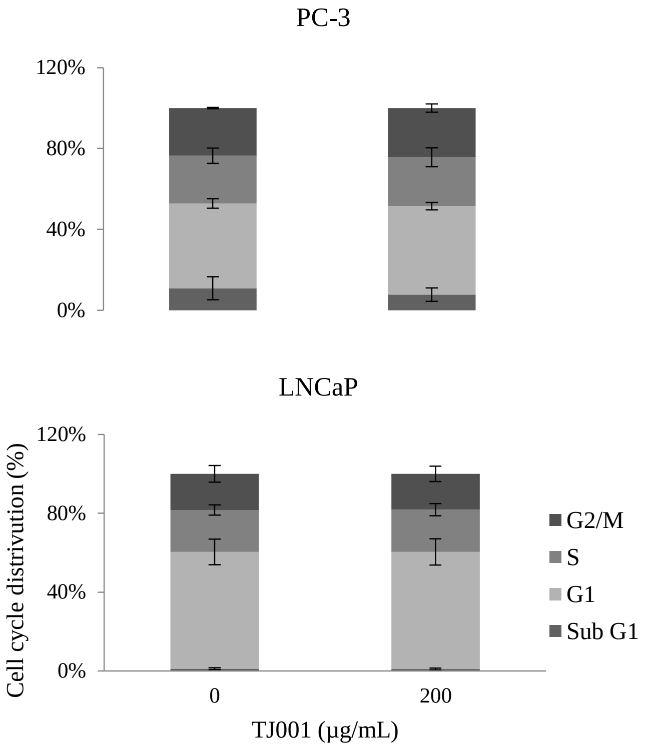
PC-3
120%
80%
40%
0%
LNCaP
120%
80%
40%
0%
Cell cycle distrivution (%)
0
200
TJ001 (μg/mL)
G2/M
S
G1
Sub G1

(b)

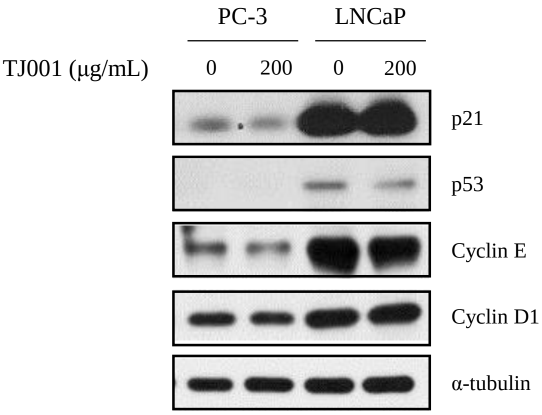
PC-3
LNCaP
TJ001 (μg/mL)
0
200
0
200
p21
p53
Cyclin E
Cyclin D1
α-tubulin

(c)

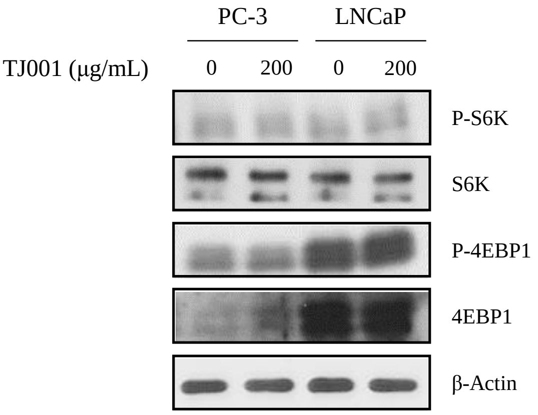
PC-3
LNCaP
TJ001 (μg/mL)
0
200
0
200
P-S6K
S6K
P-4EBP1
4EBP1
β-Actin

(d)
